# Non-coding RNAs: targets for Chinese herbal medicine in treating myocardial fibrosis

**DOI:** 10.3389/fphar.2024.1337623

**Published:** 2024-02-27

**Authors:** Minghui Wang, Maocai Yan, Liqiang Tan, Xiaona Zhao, Guoqing Liu, Zejin Zhang, Jing Zhang, Honggang Gao, Wei Qin

**Affiliations:** ^1^ School of Pharmacy, Shandong University of Traditional Chinese Medicine, Jinan, Shandong, China; ^2^ School of Pharmacy, Jining Medical University, Rizhao, Shandong, China; ^3^ Department of Nasopharyngeal Carcinoma, Sun Yat-Sen University Cancer Center, Guangzhou, Guangdong, China; ^4^ School of Pharmacy, Weifang Medical University, Weifang, Shandong, China; ^5^ School of Pharmacy, Binzhou Medical University, Yantai, Shandong, China

**Keywords:** myocardial fibrosis, Chinese herbal medicine, ncRNAs, miRNAs, lncRNAs, circRNAs

## Abstract

Cardiovascular diseases have become the leading cause of death in urban and rural areas. Myocardial fibrosis is a common pathological manifestation at the adaptive and repair stage of cardiovascular diseases, easily predisposing to cardiac death. Non-coding RNAs (ncRNAs), RNA molecules with no coding potential, can regulate gene expression in the occurrence and development of myocardial fibrosis. Recent studies have suggested that Chinese herbal medicine can relieve myocardial fibrosis through targeting various ncRNAs, mainly including microRNAs (miRNAs), long non-coding RNAs (lncRNAs), and circular RNAs (circRNAs). Thus, ncRNAs are novel drug targets for Chinese herbal medicine. Herein, we summarized the current understanding of ncRNAs in the pathogenesis of myocardial fibrosis, and highlighted the contribution of ncRNAs to the therapeutic effect of Chinese herbal medicine on myocardial fibrosis. Further, we discussed the future directions regarding the potential applications of ncRNA-based drug screening platform to screen drugs for myocardial fibrosis.

## Graphical Abstract

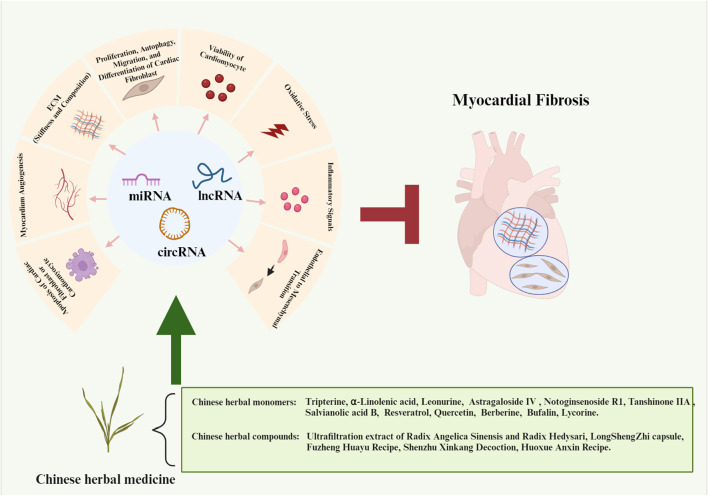

## 1 Introduction

The main pathological features of myocardial fibrosis (MF) are excessive deposition of extracellular matrix in myocardial interstitium (Espeland et al., 2018). MF is a pathological manifestation of many cardiovascular diseases, like myocardial infarction (Talman and Ruskoaho, 2016), myocarditis (Tymińska et al., 2021), coronary heart disease (He et al., 2020) and hypertension (Rai et al., 2019). For example, following myocardial infarction, a large number of cardiac fibroblasts (CFs) are activated and transdifferentiate into myofibroblasts, which has stronger contraction function and extracellular matrix synthesis ability. Then increased collagen can deposit and form scar tissue in the infarcted area to provide support for the heart (González et al., 2018). Besides myocardial infarction, pressure overload due to hypertension or aortic stenosis (Verjans et al., 2018), volume overload induced by mitral valve (Dit Beaufils et al., 2021) or aortic valve insufficiency (Fragasso et al., 2022), and the release of inflammatory factors caused by oxidative stress (Lu et al., 2022) can also result in MF. MF is the end-stage pathological manifestation of most cardiovascular diseases and is closely related to cardiac death. It affects cardiac function in various ways. On one hand, perivascular fibrosis can impact coronary blood supply, leading to myocardial ischemia, hypoxia, and even necrosis (Ytrehus et al., 2018). On the other hand, the deposition of a large number of collagen fibers results in an increase in ventricular wall hardness and a decrease in compliance, leading to reduced myocardial contraction, synchronization, and overall cardiac function (Sutton and Sharpe, 2000; Prabhu, 2005). Additionally, the deposition of collagen fibers in the matrix affects the conduction of myocardial electrical signals, easily forming reentrant rings and conduction blocks, which can result in arrhythmia (Francis Stuart et al., 2016). Therefore, studying the theraputic strategies of MF is helpful to slow down the progression of cardiovascular diseases and reduce the mortality rate of patients.

At present, the treatment of MF mainly involves two approaches: medication and surgery. Medication methods are more commonly used at the early stage of cardiovascular diseases to prevent the development of MF. These drug mainly include angiotensin-converting enzyme inhibitors (Francis Stuart et al., 2016), diuretics (Liu and Yu, 2022), β-blockers (Kobayashi et al., 2004), and anticoagulants (Oh et al., 2022). Surgical methods are used at the late stage of cardiovascular diseases, mainly including endocardial resection, atrioventricular valve repair/replacement, and heart transplantation. Endocardial resection can achieve the purpose of treatment by removing endocardial fibrous hyperplasia and calcification (Eckardt et al., 2000). Atrioventricular valve repair/replacement can repair/replace the fibrotic valve and restore its normal function (Mbanze et al., 2020). The heart transplantation can be performed when other treatments for heart problems have not worked, leading to heart failure (Srivastava and Kittleson, 2024). The aforementioned Western medicine methods offer the advantages of quick and potent effects, but their associated risks and side effects cannot be ignored. For instance, some medications may cause varying degrees of side effects, such as nausea, headaches, and liver function impairment; different patients may exhibit varied responses to the same treatment methods, leading to inconsistent treatment outcomes; some treatment methods may be relatively expensive, posing an economic burden on patients.

Chinese herbal medicine is an important historical treasure. Unlike Western medicine, Chinese herbal medicine offers the advantages of having minimal side effects in clinical applications. Prescriptions are tailored to individual patients, considering factors such as time and condition. From the perspectives of “blood stasis” and “phlegm turbidity”, Chinese herbal medicine is utilized in the treatment of MF, often in combination with compounds that promote blood circulation, remove blood stasis, and address phlegm and turbidity. Clinically, widely used formulations include Qishen Yiqi pill (Lv et al., 2021), Tongxinluo Capsule (Yin et al., 2019), Baoxin Decoction (Sun et al., 2017), Ginseng Dingzhi Decoction (Wanget al., 2022a), Huoxue Anxin Recipe (Wang et al., 2016), and others.

Non-coding RNAs (ncRNAs) are identified as RNA molecules with no coding potential (Yan and Bu, 2021). With the development of RNA sequencing technology, a large number of ncRNAs have been identified in different species and tissues. For a long time, it is generally believed that most genetic information is processed by protein-coding genes, while ncRNAs were regarded as junk nucleic acid sequences (Palazzo and Lee, 2015). In recent years, systematic analysis of cardiovascular genome and transcriptome has profoundly changed people’s understanding of ncRNAs. Numerous studies have confirmed that ncRNAs are important regulatory factors in heart development and have an inseparable relationship with the occurrence and development of most cardiovascular diseases (Qu et al., 2016). By forming complexes with RNA, DNA, or proteins, ncRNAs modulate numerous pivotal signaling pathways implicated in MF (Chen et al., 2018b; Dilmaghnai et al., 2021). Moreover, the expression changes of ncRNAs in plasma and tissues can be regarded as biomarkers for early warning and predicting prognosis of MF (Jiang et al., 2020; Ghafouri-Fard et al., 2021). As such, they represent promising future clinical targets for modulating both MF and its associated cardiovascular conditions. In recent years, studies have shown that ncRNAs are the targets of Chinese herbal medicine in the treatment of MF. In this study, we have discussed recent progress in the modulation of ncRNAs through Chinese herbal medicine for managing MF, with a focus on herbal monomers and compounds.

## 2 Mechanisms of ncRNAs in regulating gene expression

**FIGURE 1:**
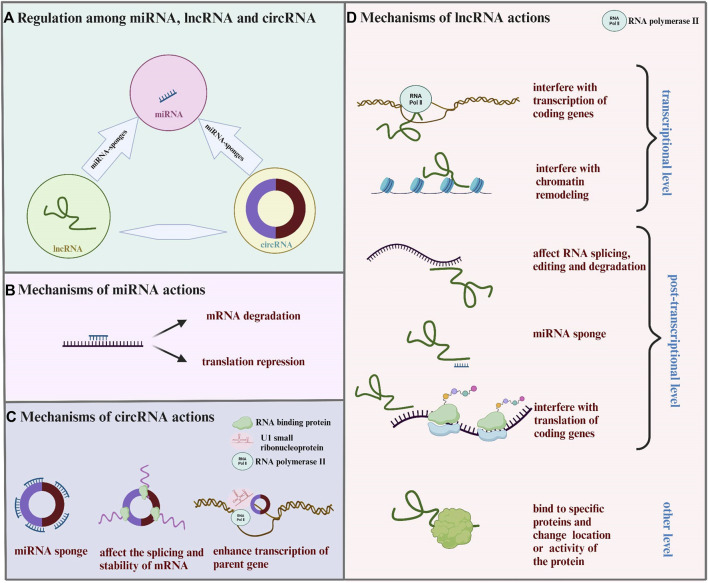
Mechanisms of controlling gene expression through ncRNAs. **(A)** LncRNA and circRNA act as miRNA sponges and affect the expression of miRNA and its target gene. **(B)** MiRNA binds to mRNA, leading to mRNA degradation or translation inhibition. **(C)** CirRNA combines with miRNA, RNA binding protein, U1 small ribonucleoprotein particles, and RNA polymerase II to affect the function of miRNA, interfere with the splicing or stability of mRNA, and enhance the transcription of parent genes. **(D)** LncRNA regulats gene expression from the transcriptional level, post-transcriptional level, and other level. Transcriptional level: lncRNA interferes with the transcription or chromatin remodeling of coding genes. Post-transcriptional level: lncRNA acts as miRNA sponges; lncRNA forms RNA double strand with mRNA, affecting mRNA splicing, editing, degradation, and translation. Other level: lncRNA binds to specific proteins, changing the location or activity of the protein.

In the past few decades, ncRNAs have been proved to regulate gene expression at multiple levels in the occurrence and development of MF, and act as new drug targets for MF treatment. These ncRNAs mainly include microRNA (miRNA), long non-coding RNA (lncRNA), and circular RNA (circRNA), of which miRNA-mediated regulation have mostly been studied and documented (Dong et al., 2019b). Intrestingly, there is also an interaction between the three ncRNAs, called competitive endogenous RNA (ceRNA) mechanism, which complicates gene regulation. The mechanisms of ncRNAs in regulating gene expression and their inter-regulations are shown in Figure 1.

### 2.1 MiRNA in regulating gene expression

MiRNA is a highly conserved gene family with a length of about 22–25 nucleotides (Davoodvandi et al., 2021). MiRNA binds to the untranslated region at the 3’ end of mRNA to inhibit mRNA transcription or translation (Mohr and Mott, 2015). Studies have shown that pri-miRNA, the primary transcription product of miRNA, is cut into hairpin precursor miRNA (pre-miRNA) by Drosha enzyme of ribonuclease 3 family. After preliminary cutting, pre-miRNA is transported from the nucleus to the cytoplasm by transporter. Then, the pre-miRNA is further cut by the combined action of Dicer enzyme of ribonuclease 3 family and dsRNA binding protein (dsRBP). Finally, the mature miRNA binds to argonaute (AGO) protein to form RNA-inducing silencing complex (RISC), leading to the interaction between the complex and the target mRNA (Leitão and Enguita, 2022). There are two ways in which miRNA regulates gene expression: when miRNA is completely complementary to mRNA, the mRNA can be directly cut; when miRNA binds with mRNA incompletely, the translation of mRNA is prevented but the stability of mRNA is not affected (Fabian et al., 2010).

Through regulating fibrosis-related factors, miRNA plays various roles in MF. For example, mir-338-3p acts as a therapeutic target in MF through fibroblast growth factor receptor 2 (FGFR2) suppression (Huang et al., 2022a). MiR-125b is critical for induction of MF by targeting p53 and Apelin mRNA (Nagpal et al., 2016). The matrix metalloproteinases (MMPs) and tissue inhibitors of MMPs (TIMPs) play crucial roles as regulators of extracellular matrix turnover and tissue remodeling, significantly influencing MF (Serraino et al., 2023). A previous study has shown that miR-146b-5p can bind to TIMP4 mRNA, regulating the balance between TIMP4 and MMP9, which is associated with atrial fibrosis (Ye et al., 2021). Additionally, MMP2 and MMP9 have been identified as potential targets for miR-29a and miR-133a (Jones et al., 2011). Connective tissue growth factor (CTGF) exerts chemotactic and mitogenic effects on fibroblasts, closely related to the occurrence and development of fibrosis in various tissues and organs (Ramazani et al., 2018). MiR-30a attenuates MF in rats by targeting CTGF (Chen et al., 2018a). Furthermore, Galectin-3 promotes the proliferation and collagen synthesis of CFs, while miR-335 inhibits MF by directly targeting this gene Sun.

### 2.2 LncRNA in regulating gene expression

Compared with miRNA, the length of lncRNA is usually longer, generally more than 200 nt, which has the similar structure with mRNA (Jha et al., 2023). Firstly, ceRNA mechanism is one of the important ways for lncRNA to regulate genes (Huang, 2018). As a natural miRNA sponge, lncRNA competes with mRNA to bind with miRNA, which affects gene silencing induced by miRNA. For example, lncRNA PFL acts as a ceRNA of let-7d to promote fibrogenesis (Liang et al., 2018), Besides ceRNA mechanism, lncRNA can also directly bind to DNA, mRNA and protein. This modulation can be categorized into three levels: transcription level, post-transcription level and other level (Li et al., 2023b). Transcriptional level means lncRNA can interfere with the transcription or chromatin remodeling of coding genes (Dykes and Emanueli, 2017). For example, lncTCF7 recruit switch/sucrose non-fermentable (SWI/SNF) complex to transcription factor 7 (TCF7) promoter region, leading to transcription of TCF7 gene (Wang et al., 2015b). Post-transcriptional level means lncRNA can influence mRNA splicing or translation (Ma et al., 2013). For example, IncRNA Safe can complementarily combine with secreted frizzled-related protein 2 (Sfrp2) mRNA to form a Safe-Sfrp2 RNA duplex to stabilize each other (Hao et al., 2019). Moreover, lncRNA can also bind to specific proteins, changing the location of the protein or regulating its activity (Guttman and Rinn, 2012). For example, lncRNA HOX transcript antisense RNA (HOTAIR) could bind with polypyrimidine tract-binding protein 1 (PTBP1) to increase the stability of Wnt5a (Tan et al., 2022).

The mechanism of regulating MF by lncRNA is complicated, with numerous lncRNAs involved in the ceRNA network to exert their functions. For example, lncRNA DANCR targets miR-758-3p to regulate proteoglycan 4 (PRG4) and the downstream Smad pathway, influencing the progression of cardiac dysfunction and fibrosis (Huang and Huang, 2023). LncRNA CFAR promotes MF via targeting miR-449a-5p to regulate the lysyl oxidase-like protein-3 (LOXL3)/mammalian target of rapamycin (mTOR) axis (Zhang et al., 2023). LncRNA TUG1 exacerbates MF in diabetic cardiomyopathy by modulating the miR-145a-5p/cofilin-2 (Cfl2) axis (Wang et al., 2023). Unlike the above mechanism, some lncRNAs regulate MF by directly decoying proteins. For example, the regulatory role of lncRNA Wisper in CFs proliferation, migration, and survival depends on its association with TIA1-related protein (Micheletti et al., 2017). LncRNA MetBil directly binds to methyltransferase like 3 (METTL3) protien to regulate its expression in ubiquitin-proteasome pathway, thereby regulating the expression of the methylated fibrosis-associated genes in ischemia-induced MF (Zhuang et al., 2023).

### 2.3 CircRNA in regulating gene expression

CircRNA has a closed loop structure without 5′cap and 3′polyA tail, and the average length of circRNA in human body is about 500 nt (Dragomir and Calin, 2018). CircRNA contains abundant miRNA binding sites and can be used as a sponge of miRNA (Salmena et al., 2011). For example, circRNA-005647 has a binding site with miR-27b-3p and inhibits the binding of miR-27b-3p with fibrosis-related genes (Yuan et al., 2019). In addition to ceRNA mechanism, circRNA can affect the splicing and stability of mRNA by binding to RNA binding protein (RBP). For example, circFndc3b enhances the expression and signal transduction of vascular endothelial growth factor-A (VEGF-A) by interacting with RBP fused in sarcoma (FUS) (Garikipati et al., 2019). What’s more, circRNA can also bind to the promoter region and enhance the transcription of its parent gene by interacting with U1 small ribonucleoprotein particles and RNA polymerase II (Lun et al., 2023).

The mechanisms currently reported for circRNA in regulating MF mainly involve acting as a ceRNA, interacting with proteins, and encoding proteins. For example, circSMAD4 promotes MF by acting as a sponge against miR-671-5p (Jeong et al., 2023). CircYap directly binds to tropomyosin-4 (TMP4) and gamma-actin (ACTG) to make the interaction between the two proteins more stable, resulting in the inhibition of actin polymerization and subsequent MF (Wu et al., 2021). Circ_0036176 has the ability to encode a protein containing 208 amino acids named Myo9a-208, which mediates the inhibitory effect of circ_0036176 on the proliferation of CFs (Guo et al., 2022).

## 3 Chinese herbal medicine relieves MF through regulating ncRNAs

**FIGURE 2:**
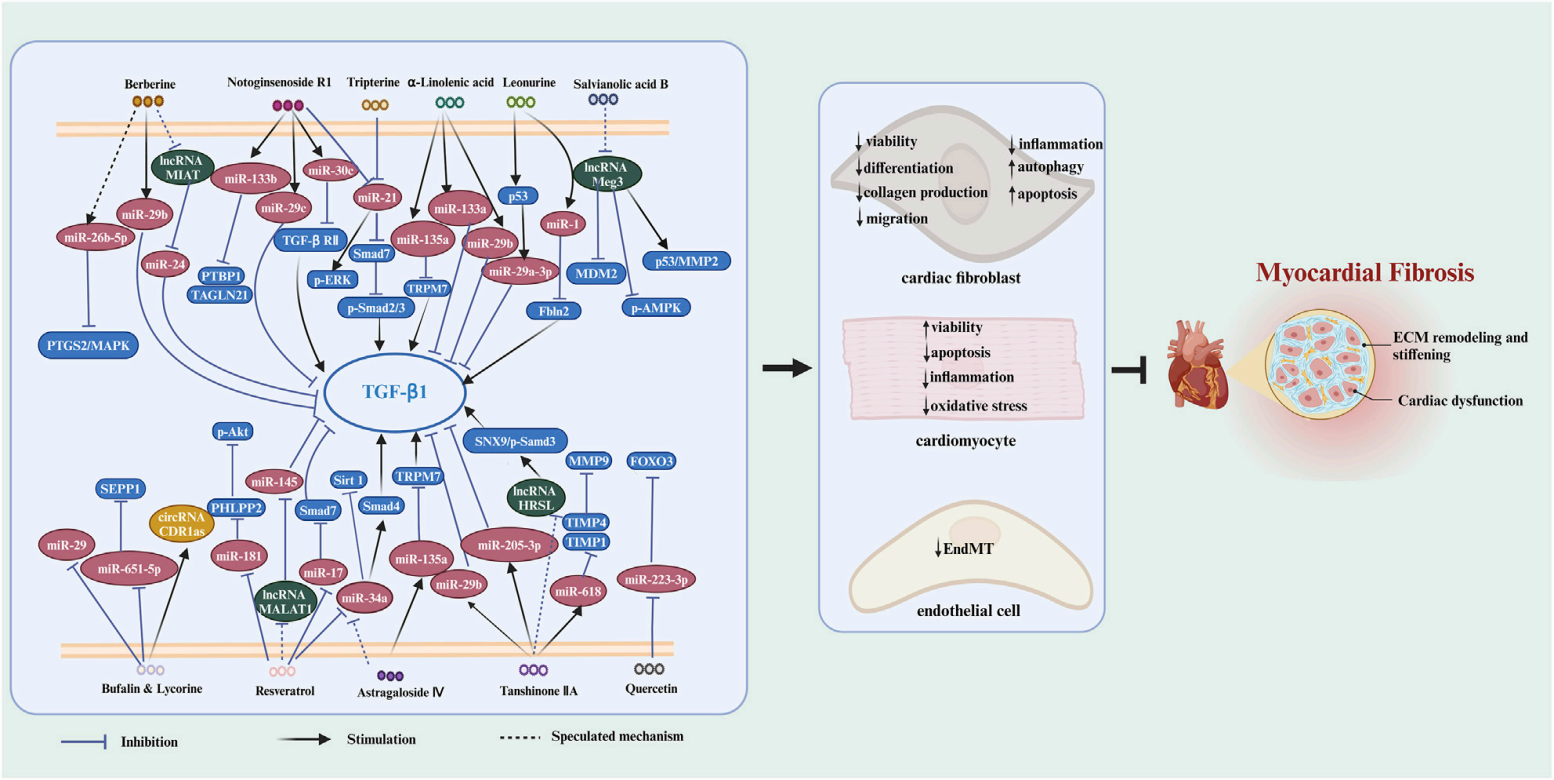
The signaling pathways of Chinese herbal monomers in treating MF by interfering with ncRNAs. Chinese herbal monomers regulate ncRNAs to inhibit the viability, differentiation, migration, inflammation, and collagen production of cardiac fibroblasts while triggering apoptosis and autophagy in these cells. Furthermore, these herbal monomers can also inhibit inflammation, oxidative stress, and apoptosis in cardiomyocytes and enhance their viability through ncRNA regulation. Additionally, EndMT can also be inhibited by Chinese herbal monomers in a ncRNAs-dependent way. These mechanisms collectively contribute to the ultimate inhibition of myocardial fibrosis. The blank arrow indicates promotion, the blue T-shaped arrow indicates inhibition, and the black dotted arrow indicates the presumed regulatory function.

**FIGURE 3:**
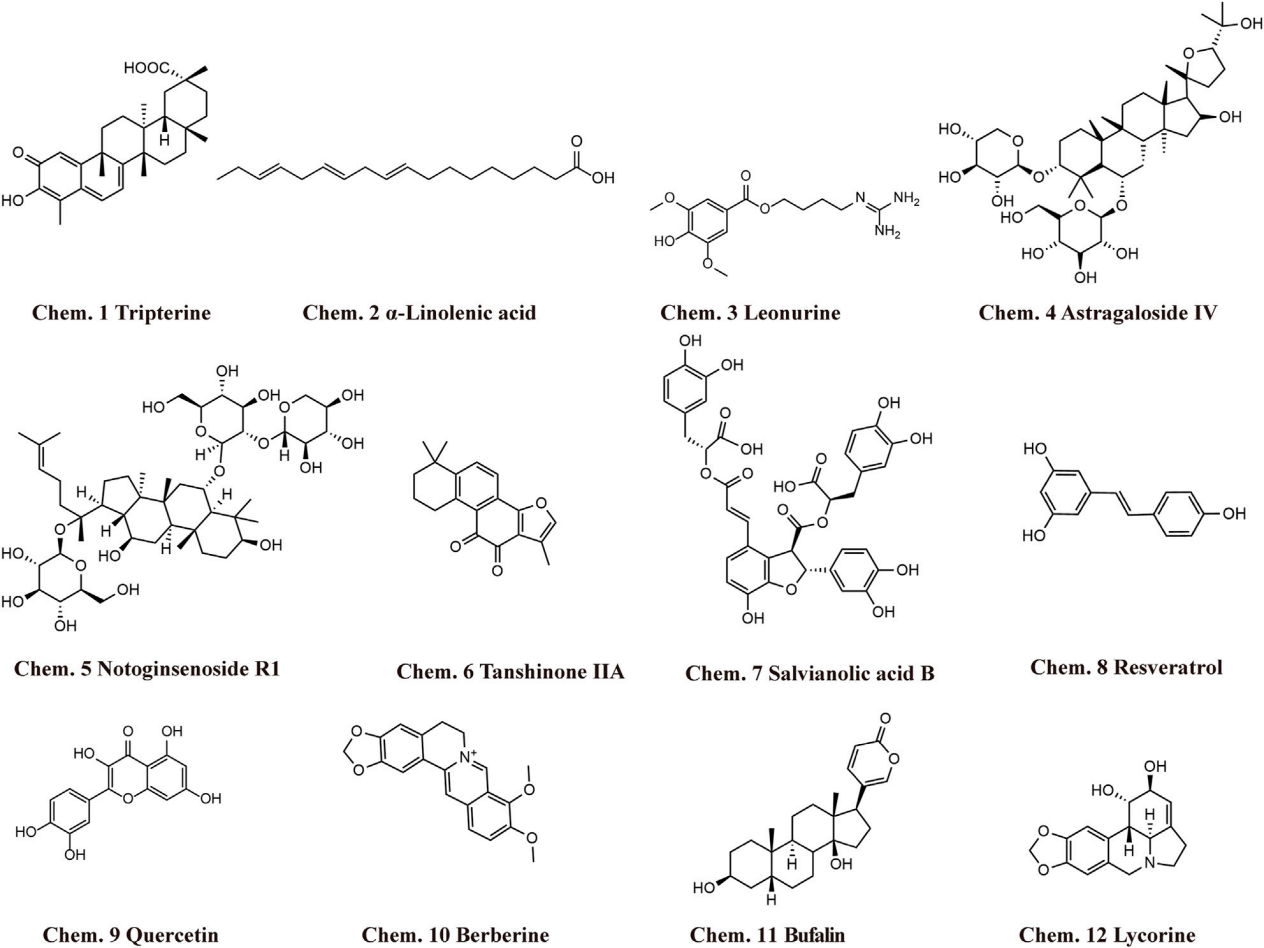
Structural formula of Chinese herbal monomers that exhibit anti-MF activity by interfering with ncRNAs.

**FIGURE 4:**
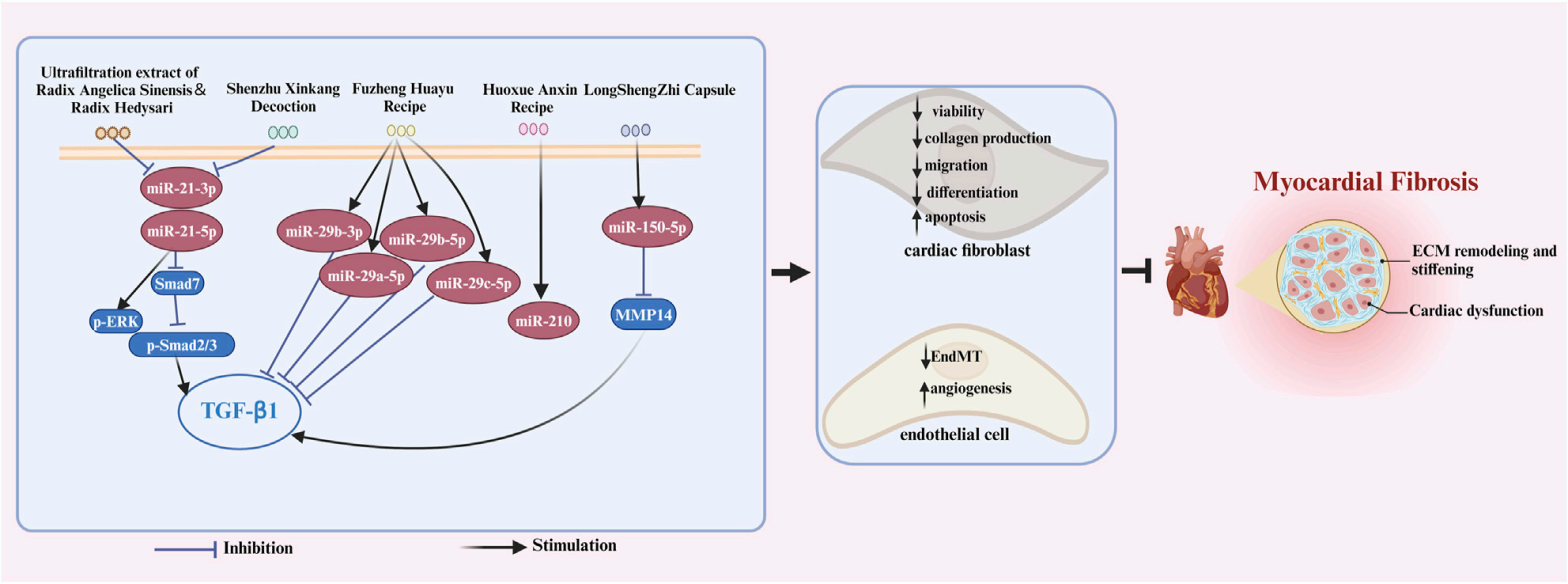
The signaling pathways of Chinese herbal compounds in treating MF by interfering with ncRNAs. Chinese herbal compounds regulate ncRNAs to inhibit the viability, differentiation, migration, and collagen production of cardiac fibroblasts while triggering apoptosis in these cells. Moreover, these herbal compounds can also inhibit EndMT and promote myocardium angiogenesis through ncRNAs regulation. These mechanisms collectively contribute to the ultimate inhibition of myocardial fibrosis. The blank arrow indicates promotion, the blue T-shaped arrow indicates inhibition.

Chinese herbal medicine has the characteristics of wide sources and small side effects, thus having a unique advantage in treating human diseases including MF. Recent studies have suggested that Chinese herbal medicine can exert anti-MF effects by regulating ncRNAs. 12 kinds of Chinese herbal monomers and 5 kinds of Chinese herbal compounds have been shown to treat MF by interfering with ncRNAs. The herbal monomers include tripterine, α-linolenic acid, leonurine, astragaloside IV, notoginsenoside R1, tanshinone IIA, salvianolic acid B, resveratrol, quercetin, berberine, bufalin, and lycorine. The pharmacological action and mechanisms of these Chinese herbal monomers are summarized below in Figure 2. The chemical structural formulas of Chinese herbal monomers are shown in Figure 3. The Chinese herbal compounds include trafiltration extract of radix angelica sinensis and radix hedysari, Longshengzhi capsule, Fuzheng Huayu Recipe, Shenzhu Xinkang Decoction, and Huoxue Anxin Recipe. The pharmacological action and mechanisms of these Chinese herbal compounds are summarized below in Figure 4.

### 3.1 Chinese herbal monomers and their targeting ncRNAs in treating MF

#### 3.1.1 Tripterine

Chinese herb *Tripterygium wilfordii* Hook. F. has the effects of dispelling dampness, relieving swelling and pain, and resisting inflammation (Zeng et al., 2024). Triptolide and tripterine are the two most active components in the extract of *Tripterygium wilfordii* Hook. F. In recent years, it has been found that tripterine can inhibit CFs viability and collagen production by down-regulating the expression of miR-21, the activator of ERK signaling pathway, leading to relieved MF and cardiac dysfunction (Cheng et al., 2016). In fact, miR-21 has been found to be a pro-fibrotic factor in various animal models, targeting multiple fibrotic pathways and promoting MF. For example, in a mouse model of myocardial infarction, miR-21 promotes CF activation and MF via TGF-β/Smad7 signaling pathway (Yuan et al., 2017). In another diabetes-induced MF mouse model, silencing miR-21 inhibits high glucose-induced endothelial to mesenchymal transition (EndMT) (Li et al., 2020). These results suggest that the anti-fibrotic effect of tripterine is mediated by miR-21 and the downstream ERK, TGF-β, and EndMT signaling pathways.

#### 3.1.2 α-Linolenic acid (ALA)

Flaxseed oil is extracted from *Linum usitatissimum* L., and is rich in omega-3 fatty acids such as ALA. It is usually used to lower cholesterol, resist atherosclerosis, and reduce heart load (Prasad et al., 2020). A study has shown that in a rat model of myocardial infarction, flaxseed oil exerts cardioprotective effect and decreases collagen deposition via selectively up-regulaing the expression of miR-133a, miR-135a, and miR-29b. The author speculated this effect may be attributed to ALA component in the flaxseed oil (Parikh et al., 2020). Previous studies have shown that the three miRNAs are all anti-fibrotic miRNAs. MiR-133a reduces MF by suppressing transforming growth factor-β1 (TGF-β1) signaling in an acute myocardial infarction model (Yu et al., 2019). MiR-135a could target transient receptor potential melastatin 7 (TRPM7) to inhibit the activation of TGF-β/Smads pathway, thus relieving MF (Wei et al., 2020). MiR-29b inhibits many genes involved in extracellular matrix formation and fibrosis, such as Col1a1, Col1a2, Col3a1, fibrillin 1, Elastin, and TGF-β1 (Rooij et al., 2008; Zhang et al., 2014).

#### 3.1.3 Leonurine

Leonurine, an alkaloid extracted from *leonurus japonicus* Houtt., has been shown to have various pharmacological effects including protecting myocardial ischemia-reperfusion injury, resisting blood platelet aggregation, reducing blood viscosity, promoting angiogenesis, lowering blood pressure, and inducing diuresis (Huang et al., 2021). Recent studies have shown that leonurine can treat MF induced by isoproterenol by upregulating miR-1, which could directly target Fibullin-2 (Fbln2) to reverse cardiac remodeling (Karakikes et al., 2013; Lu et al., 2018). In addition, evidence has shown that all mature miR-29 family members in post-myocardial infarction tissues are downregulated (Wang et al., 2021). Leonurine treatment can significantly upregulate the expression of miR-29a-3p and downregulate its target proteins including TGF-β, Col3a1, and Col1a1, to attenuate fibrosis and cardiac remodeling (Wang et al., 2021). What’s more, a study confirmed that leonurine can also promote apoptosis of CFs through regualting miR-29a-3p (Xi et al., 2023). These evidences suggest that the anti-fibrotic effect of leonurine may be mediated by miR-1 and miR-29a-3p.

#### 3.1.4 Astragaloside IV

Astragaloside IV is one of the best bioactive components from the root of *Astragalus membranaceus* (Fisch.) Bunge (Jing et al., 2021). Studies have shown that astragaloside IV inhibits MF by up-regulating the expression of miR-135a which targets transient receptor potential melastatin 7 (TRPM7) (Wei et al., 2020). Moreover, in an experiment of high glucose-induced injury of cardiomyocytes, it was found that astragaloside IV could reduce the expression of miR-34a (Zhu et al., 2019). According to previous studies, inhibition of miR-34a can treat MF by inhibiting cardiomyocytes apoptosis (Dong F. et al., 2019a). Therefore, it can be speculated that astragaloside IV may play an anti-fibrotic role through repressing cardiomyocytes apoptosis by decreasing miR-34a.

#### 3.1.5 Notoginsenoside R1

Panax notoginseng saponins are the main component of the roots of *Panax notoginseng* (Burk.) F. H. Chen. Panax notoginseng saponins have the effects of promoting blood circulation, removing blood stasis, and dredging collaterals, which are commonly used to treat coronary heart disease and blood stasis syndrome (Jiao et al., 2021; Yang et al., 2022). Notoginsenoside R1 is the primary active component of Panax notoginseng saponins. It was found that notoginsenoside R1 inhibits isoproterenol-induced MF through the intervention of miRNA-mRNA regulatory network, among which the expression level of miR-21 decreased, while miR-29c, miR-30c and miR-133b increased (Ning, 2016). These miRNAs have all been proven to be fibrosis-related miRNAs. It has been shown that miR-21 activates ERK signaling pathway, EndMT and TGF-β signaling pathway to promots MF (Cheng et al., 2016; Yuan et al., 2017; Li et al., 2020). MiR-29c has been proved to target multiple fibroisis-related genes including Col1a1, Col1a2, Col3a1 and Col5a1, fibrllin 1 and TGF-β1 (Liu et al., 2017) MiR-30c inhibits the proliferation, differentiation, migration and collagen production of CFs by targeting TGF-βRII (Xu et al., 2018); MiR-133b has also been reported to alleviate doxorubicin-induced cardiomyocyte apoptosis and MF by targeting PTBP1 and transgelin 2 (TAGLN2) (Li et al., 2021). Therefore, notoginsenoside R1 can target multiple fibrosis-related miRNA and can be regarded as attractive anti-fibrotic candidate medicine.

#### 3.1.6 Tanshinone IIA

Tanshinone IIA is extracted from the dried root and rhizome of *Salvia miltiorrhiza* Bunge, which exerts a wide range of cardioprotective effects in the diseases like myocardial infarction, myocardial ischemia-reperfusion injury, myocardial hypertrophy, atherosclerosis, and cardiomyopathy (Zhang et al., 2019; Yang et al., 2020). Recent studies have shown that tanshinone IIA can relieve MF through up-regulating miR-29b (Yang et al., 2015), miR-205-3p (Qiao et al., 2021), and miR-618 (Yan et al., 2022) expressions. MiR-29b and miR-205-3p could downregulate the expression of TGF-β1, Col1a1, and Col3a1, so as to resist MF following myocardial infarction (Yang et al., 2015; Qiao et al., 2021). MiR-618 could inhibit the expression of TIMP1 and TIMP4 to mediate the anti-fobrotic effects of tanshinone IIA on CFs (Yan et al., 2022). In addition, it has been found that tanshinone IIA can inhibit fibroblast proliferation by down-regulating lncRNA human-specific regulatory loci (HSRL) in skin hypertrophic scar tissue. HSRL could promote the expression of sorting connexin 9 (SNX9) and strengthen its interaction with p-Smad3, thus activating TGF-β signaling (Shi et al., 2020). Therefore, inhibiting HSRL may also mediate the anti-fibrotic effect of tanshinone IIA.

#### 3.1.7 Salvianolic acid B

Salvianolic acid B is also derived from the root and rhizome of *Salvia miltiorrhiza* Bunge. A study showed that lncRNA maternally expressed gene 3 (Meg3), mainly expressed in CFs, is a new promotor of MF. Silencing Meg3 prevents MMP2 production, leading to the decreased MF and improved cardiac function (Piccoli et al., 2017). In cardiomyocytes of oxygen and glucose deprivation (OGD), salvianolic acid B was reported to represses Meg3 expression, which influences murine double minute 2 (MDM2)/p53 and AMP-activated Protein Kinase (AMPK) signalling pathways, leading to incresed viability and reduced apoptosis of cardiomyocytes (Yang et al., 2019a). Therefore, it is speculated that salvianolic acid B may have the cardioprotective effect of inhibiting MF by down-regulating Meg3, but more researches are needed to test this hypothesis.

#### 3.1.8 Resveratrol

Resveratrol is an active polyphenol, derived from many herbal medicines, such as *Morus alba* L., *Polygonum cuspidatum* Sieb. et Zucc., and *Rubus idaeus* L. Resveratrol is proved to have anti-bacterial, anti-inflammatory and immunomodulatory effects (Malaguarnera, 2019). In cardiovascular system, it exerts protective effects on atherosclerosis, myocardial infarction, and heart failure (Raj et al., 2021). Recent studies showed that resveratrol can inhibit proliferation and induce cell death of CFs (Lieben Louis et al., 2019). A study assessed the impact of resveratrol on microRNAs linked to MF, and found resveratrol inhibits the expressions of miR-17, miR-34a and miR-181a in TGF-β1-induced CFs (Zhang et al., 2018). Overexpression of miR-17 decreases Smad7 expression level, indirectly promotes TGF-β1 signaling (Zhang et al., 2018). MiR-34a activates TGF-β1 signaling through increasing Smad4 expression (Huang et al., 2014). MiR-181a suppresses the expression of PH domain leucine-rich repeat protein phosphatase 2 (PHLPP2) and subsequently activates AKT signaling, leading to enhanced proliferation of keloid fibroblast cells (Rang et al., 2016). In addition, resveratrol was reported to inhibit the expression of lncRNA metastasis associated lung adenocarcinoma transcript 1 (MALAT1), which could act as a ceRNA of miR-145 and promote MF progression (Yang et al., 2019b; Huang et al., 2019). Therefore, downregulation of MALAT1 may be one of the potential mechanisms of resveratrol in treating MF. It is evident that resveratrol, with its multiple ncRNA targets, holds significant promise for the management of MF.

#### 3.1.9 Quercetin

Quercetin is a kind of flavonol that widely exists in flowers, leaves and fruits of many plants, such as *Sophora japonica* L, *Asparagus officinalis* L., and *Acanthopanax senticosus* (Rupr.et Maxim.) Harms. Previous studies have shown that quercetin restrains the level of fibrotic proteins including TGF-β1, α-SMA, Col1a1, and Col3a1 in heart tissue of myocardial infarction model (Albadrani et al., 2021). Studies are progressively uncovering that the promotion of CFs autophagy can effectively inhibit MF and enhance cardiac function (Wang et al., 2015a). Quercetin was found to prevent isoprenaline-induced MF by increasing autophagy of CFs via decreasing miR-223-3p and increasing forkhead Box O3 (FOXO3) (Hu et al., 2021).

#### 3.1.10 Berberine

Berberine is a quaternary ammonium alkaloid contained in the rhizome of *Coptis chinensis* Franch. and has various cardiovascular protective effects, such as anti-heart failure, anti-arrhythmia, and lowering cholesterol effects (Pagliaro et al., 2015; Zhao et al., 2020). It was found that berberine relieves hypertension-induced MF by increasing the expression of miR-29b and decreasing its targets Col1a1 and Col3a1 (Zheng et al., 2020). Moreover, in a myocardial ischemia-reperfusion mouse model, the protective effect of berberine is exerted by inducing miR-26b-5p and inhibiting its downstream PTGS2 and MAPK members, which results in the increased viability, and decresed apoptosis, inflammatory, and oxidative stress in cardiomyocytes (Jia et al., 2022). Since the injury of the cardiomyocytes is the major causes of MF, it is speculated that miR-26b-5p may be a potential target of berberine in the treatment of MF. In addition, berberine was also proved to inhibit lncRNA myocardial infarction-associated transcript (MIAT) to improve myocardial hypertrophy (Zeng et al., 2019). Since MIAT is a pro-fibrotic lncRNA governing MF by down-regulating miR-24 and up-regulating Furin and TGF-β1 (Qu et al., 2017), we can infer that berberine may have the effect to improve MF by inhibiting MIAT.

#### 3.1.11 Bufalin and lycorine

Bufalin comes from dried *toad*, while lycorine is an alkaloid found in the bulb of *Lycoris radiata* (L'Hér.) Herb. Recently, high-throughput natural compound library screening identified bufalin and lycorine to be effective anti-fibrotic molecules in hypertension-induced MF mouse model (Schimmel et al., 2020). The study found the level of miR-671-5p is reduced after treatment of CFs with bufalin and lycorine, which leads to the increased expression of anti-fibrotic protein selenoprotein P1 (SEPP1) (Schimmel et al., 2020). Another study discovered that bufalin and lycorine can reduce the expression of miR-29 while increasing the expression of circRNA CDR1as. This, in turn, leads to a decrease in the infarction area and fibrotic area in a heart failure pig model (Mester-Tonczar et al., 2020). Consequently, the anti-fibrotic impact of bufalin and lycorine can be ascribed to the reduction of miR-671-5p and miR-29 levels and the elevation of CDR1as levels.

### 3.2 Chinese herbal compounds and their targeting ncRNAs in treating MF

#### 3.2.1 Ultrafiltration extract of radix angelica sinensis and radix hedysari

Radix Angelica Sinensis is usually used in combination with other drugs to treat cardiovascular diseases such as radiation-induced heart disease, atherosclerosis, and ischemic heart disease (Huang et al., 2022b; Li et al., 2023a; Yuan et al., 2023), while Radix Hedysari has been proved to have significant effects in treating non-alcoholic fatty liver disease (Sun et al., 2014). In a rat model of X-irradiation-induced MF, ultrafiltration extract derived from dried root of Radix Angelica Sinensis and Radix Hedysari downregulates miR-21-3p and miR-21-5p, inducing the apoptosis of CFs and alleviating MF (Ma et al., 2019). It has been shown that miR-21 activates ERK signaling pathway, EndMT, and TGF-β signaling pathway to promote MF (Cheng et al., 2016; Yuan et al., 2017; Li et al., 2020). Therefore, the Radix Angelica Sinensis and Radix Hedysari ultrafiltration extract may be developed as a medical countermeasure for the mitigation of radiation-induced MF.

#### 3.2.2 LongShengZhi capsule

Buyang Huanwu Decoction is a famous herbal prescription that has been used to treat stroke for centuries (Gao et al., 2021). Previous studies have shown that Buyang Huanwu Decoction can alleviate MF (Wang et al., 2022b). The compatible components of LongShengZhi capsule are similar to those of Buyang Huanwu Decoction, and this is referred to as the modern application of Buyang Huanwu Decoction. A study found that LongShengZhi capsule attenuates Angiotensin II-induced cardiac hypertrophy and fibrosis in rats. Mechanically, Longshengzhi capsule up-regulats miR-150-5p to target MMP14 in CFs, leading to reduced cardiac remodeling (Gu et al., 2022).

#### 3.2.3 Fuzheng Huayu Recipe

Fuzheng Huayu Recipe, a traditional Chinese herbal prescription, is often used in China to treat fibrosis (Wang et al., 2010; Sun et al., 2022). Recently, a study has suggested that Fuzheng Huayu Capsule inhibits myocardial infarction-induced MF by facilitating the expression of miR-29b-3p, miR-29a-5p, miR-29b-5p, and miR-29c-5p (Qi et al., 2019). It has been shown that miR-29 family are all anti-fibrotic factors with the effect of inhibiting TGF-β1 signaling and its downstream targets, resulting in reduced proliferation and collagen production of CFs (Rooij et al., 2008; Zhu et al., 2013; Zhang et al., 2014). Therefore, miR-29 family are the key mediators for the anti-fibrotic effect of Fuzheng Huayu Recipe.

#### 3.2.4 Shenzhu Xinkang Decoction

Shenzhu Xinkang Decoction is a representative prescription for the treatment of chronic heart failure and fibrosis (Zhao et al., 2023). EndMT has been shown to contribute to cardiac fibrosis (Zeisberg et al., 2007) and Shenzhu Xinkang Decoction was proved to inhibit EndMT to play an anti-fibrotic role. The possible mechanism is related to the downregulation of miR-21 level and inhibition of PTEN/PI3K/AKT pathway (Zhai et al., 2022).

#### 3.2.5 Huoxue Anxin Recipe

Huoxue Anxin Recipe is a novel formula of Chinese herbal medicine that has a good cardioprotective effect, such as promoting myocardial angiogenesis, exhibiting anti-oxidative stresses activity, and improving cardiac function during myocardial infarction (Zhang et al., 2012; Zhang et al., 2013; Wang et al., 2016). A recent study has demonstrated that Huoxue Anxin Recipe could reduce the infarction area, alleviate fibrosis, and improve the cardiac function of myocardial infarction rats, which is mainly attributed to enhanced angiogenesis by upregulation of miR-210 and VEGF (Wang et al., 2016).

## 4 Conclusion and perspective

The targeted relationship between Chinese herbal medicine and ncRNAs are hot spots in current research, which opens up a new avenue for exploring the mechanism of Chinese herbal medicine in prevention and treatment of cardiovascular diseases. This review discussed 12 kinds of Chinese herbal monomers and 5 kinds of Chinese herbal compounds which have been shown to treat MF by interfering with ncRNAs (Table 1). Through targeting ncRNAs, mainly including miRNA, lncRNA and circRNA, those herbal medicine relieves MF by inhibiting the proliferation/activation/inflammation of CFs, increasing apoptosis/autophagy of CFs, inhibiting apoptosis/inflammation/oxidative stress of cardiomyocytes, increasing viability of cardiomyocytes, repressing EndMT, and promoting myocardium angiogenesis.

**TABLE 1:** Mechanisms of Chinese herbal medicine in treating MF by regulating ncRNAs.

Herbal medicine	ncRNAs	Direct ncRNAs' targets	Biological function	Refrences
Tripterine	miR-21↓	Smad7	↓CFs viability, differentiation, migration, and collagen production	Cheng et al. (2016), Yuan et al. (2017), Li et al. (2020)
			↓EndMT process	
α-Linolenic acid	miR-29b↑	TGF-β1	↓CFs differentiation and collagen production	Rooij et al. (2008), Zhang et al. (2014), Parikh et al. (2020)
		Col1a1		
		Col1a2		
		Col3a1 fibrillin 1		
		Elastin		
	miR-133a↑	?	↓CFs differentiation and collagen production	Yu et al. (2019), Parikh et al. (2020)
	miR-135a↑	TRPM7	↓CFs viability, differentiation, and collagen production	Parikh et al. (2020), Wei et al. (2020)
Leonurine	miR-1↑	Fbln2	↓CFs collagen production	Karakikes et al. (2013), Lu et al. (2018)
	miR-29a-3p↑	TGF-β1	↓CFs viability, differentiation, migration, and collagen production	Wang et al. (2021), Xi et al. (2023)
		Col3a1	↑CFs apoptosis	
		Col1a1		
Astragaloside IV	miR-135a↑	TRPM7	↓ CFs viability, differentiation, and collagen production	Wei et al. (2020)
	*miR-34a↓	Sirt1	↓CMs apoptosis	Dong et al. (2019a), Zhu et al. (2019)
Notoginsenoside R1	miR-21↓	Smad7	↓CFs viability, differentiation, migration, and collagen production	Cheng et al. (2016); Ning (2016), Yuan et al. (2017), Li et al. (2020)
			↓EndMT process	
	miR-29c↑	?	↓CFs differentiation and collagen production	Ning (2016); Liu et al. (2017)
	miR-30c↑	TGF-β RII	↓CFs viability, differentiation, migration, and collagen production	Ning (2016); Xu et al. (2018)
	miR-133b↑	PTBP1	↓CFs collagen production	Ning (2016); Li et al. (2021)
		TAGLN2	↓CMs apoptosis	
Tanshinone IIA	miR-29b↑	?	↓CFs differentiation and collagen production	Yang et al. (2015)
	miR-205-3p↑	TGF-β1	↓CFs collagen production	Qiao et al. (2021)
	miR-618↑	TIMP1	↓CFs viability, differentiation, and collagen production	Yan et al. (2022)
		TIMP4		
	*lncRNA HSRL↓	SNX9	↓CFs viability, differentiation, and collagen production	Shi et al. (2020)
Salvianolic acid B	*lncRNA Meg3↓	p53	↓CFs collagen production	Piccoli et al. (2017), Yang et al. (2019a)
			↓CMs apoptosis	
			↑CMs viability	
Resveratrol	miR-17↓	Smad7	↓CFs viability and collagen production	Zhang et al. (2018)
	miR-34↓	Smad4	↓ CFs viability, differentiation, migration, and collagen production	Huang et al. (2014), Zhang et al. (2018)
	miR-181a↓	PHLPP2	↓CFs viability	Rang et al. (2016), Zhang et al. (2018)
	*lncRNA MALAT1↓	miR-145	↓CFs viability, differentiation, and collagen production	Yang et al. (2019b), Huang et al. (2019)
Quercetin	miR-223-3p↓	FOXO3	↑CFs autophagy	Hu et al. (2021)
			↓CFs viability and collagen production	
Berberine	*miR-26b-5p↑	?	↑CMs viability	Jia et al. (2022)
			↓CMs apoptosis, inflammation, and oxidative stress	
	miR-29b↑	?	↓CFs collagen production	Zheng et al. (2020)
	*lncRNA MIAT↓	miR-24	↓CFs viability and collagen production	Qu et al. (2017), Zeng et al. (2019)
Bufalin and Lycorine	miR-29↓	?	↓CFs collagen production	Mester-Tonczar et al. (2020)
	circRNA CRD1as↑	?		
	miR-671-5p↓	SEPP1	↓CFs differentiation, collagen production, and inflammation	Schimmel et al. (2020)
Ultrafiltration extract of Radix Angelica Sinensis and Radix Hedysari	miR-21-3p↓	Smad7	↓CFs viability, differentiation, migration, and collagen production	Cheng et al. (2016), Yuan et al. (2017), Ma et al. (2019), Li et al. (2020)
	miR-21-5p↓		↑CFs apoptosis	
			↓EndMT process	
LongShengzhi capsule	miR-150-5p↑	MMP14	↓CFs collagen production	Gu et al. (2022)
Fuzheng Huayu Recipe	miR-29b-3p↑	?	↓CFs collagen production	Qi et al. (2019)
	miR-29a-5p↑			
	miR-29b-5p↑			
	miR-29c-5p↑			
Shenzhu Xinkang Decoction	miR-21↓	?	↓EndMT process	Zhai et al. (2022)
Huoxue Anxin Recipe	miR-210↑	?	↑Myocardium angiogenesis	Wang et al. (2016)

(Marked with * is the speculated mechanism).

Abbreviations: CMs, cardiomyocytes; CFs, cardiac fibroblasts; circRNA, circular RNA; EndMT, endothelial to mesenchymal transition; Fbln2, Fibullin-2; FOXO3, forkhead box O3; lncRNA, long noncoding RNA; MALAT1, metastasis associated lung adenocarcinoma transcript 1; Meg3, maternally expressed gene 3; MF, myocardial fibrosis; miRNA, microRNA; MIAT, myocardial infarction-associated transcript; MMP14, matrix metalloproteinase 14; PHLPP2, PH, domain leucine-rich repeat protein phosphatase 2; PTBP1, polypyrimidine tract-binding protein 1; SEPP1, selenoprotein P1; SNX9, sorting connexin 9; TAGLN2, transgelin 2; TIMP1, tissue inhibitors of matrix metalloproteinase 1; TIMP4, tissue inhibitors of matrix metalloproteinase 4; TRPM7, transient receptor potential melastatin 7.

At present, however, several challenges persist in the investigation of ncRNAs’ role in the anti-MF effect of Chinese herbal medicine: (1) The regulatory effect of Chinese herbal medicine on ncRNAs is primarily validated in animal and cell models, and it is not yet guaranteed whether these effects still exist in complex human bodies. (2) The most reported ncRNA targets of Chinese herbal medicine are miRNAs. Whether lncRNA, circRNA, and other ncRNAs act as key mediators of Chinese herbal medicine’s effect has not been explored sufficiently. (3) We noted that some Chinese herbal medicine could target ncRNAs known to be associated with MF. However, there is currently no direct evidence to support the idea that these herbal medicines can relieve MF by modulating these ncRNAs. Further experiments are required to validate these scientific hypotheses.

NcRNAs have been implicated in various diseases and serve as key targets for disease treatment. However, the clinical transformation of RNA-based therapies is hindered by problems related to specificity, delivery and tolerance. Specificity problems indicate undesirable targeting effects caused by uptake of cells other than the target cells, and off-target effects caused by sequence similarity or overdose to a level much higher than endogenous expectations (Sledz and Williams, 2004; Yu et al., 2020). In addition, there is a lack of delivery vectors suitable for delivering ncRNAs to target organs and cell types (Krieg, 2011). What’s more, natural RNA molecules are highly susceptible to enzymatic degradation by serum and cellular RNases. Notably, both single-stranded and double-stranded RNAs can trigger the body’s viral defense system via pathogen-associated molecular patter (PAMP) receptors (Kumar et al., 2011). Due to these reasons, RNA-based therapies are often lack of efficiency in clinical trials. Therefore, targeting specific ncRNAs with small molecules displays potential as a therapeutic approach for disease treatment. Developing effective tools to screen small molecules against particular ncRNAs is very important and urgent. Recently, a study published in Nature has introduced an innovative technique for screening small molecules that bind to ncRNAs. They devised an unbiased screen based on affinity-selection mass spectrometry to identify reversibly binding ability between lncRNA Xist and 50,000 compounds. They found 20 analogues that has similar structure with the one positive hit and finally obtained one positive compound X1 that can effectively reguate the function of Xist (Aguilar et al., 2022). This research broadens the scope of ncRNA pharmaceutical field, which will enable the development of RNA-targeting drugs by high-throughput and large-scale screening methods. Therefore, by utilizing this screening system with MF-related ncRNAs as binding targets, it holds the promise of identifying a greater number of anti-MF drugs, including Chinese herbal medicine. For example, it has been shown that miR-21 activates the ERK signaling pathway, EndMT, and the TGF-β signaling pathway to promote MF (Cheng et al., 2016; Yuan et al., 2017; Li et al., 2020). Based on the preceding statement, miR-21 has been proven to be a target for many Chinese herbal medicines in combating MF. Tripterine, Notoginsenoside R1, Shenzhu Xinkang Decoction, and the ultrafiltration extract of Radix Angelica Sinensis and Radix Hedysari can all alleviate MF by inhibiting miR-21. Therefore, miR-21 may serve as a binding target for screening anti-MF drugs.

In conclusion, it is clear in this emerging field that ncRNAs appear to be important players in mediating Chinese herbal medicine’s effect in various diseases. Indeed, as we have reviewed, ncRNAs interact with fibrosis-related genes and signalling pathways, making them a pivotal bridge in mediating the therapeutic effect of Chinese herbal medicine on MF. Furthermore, ncRNAs represent promising clinical drug targets and establishing an anti-MF drug screening platform based on ncRNAs to screen drugs including Chinese herbal medicine represents a challenging but promising field for future drug development in cardiovascular diseases.

## Figures and Tables

**Table:** 

**AGO**	Argonute
**ALA**	α-linolenic acid
**AMPK**	AMP-activated protein kinase
**ACTG**	gamma-actin
**ceRNA**	competitive endogenous RNA
**Cfl2**	cofilin-2
**CFs**	cardiac fibroblasts
**circRNA**	circular RNA
**CTGF**	connective tissue growth factor
**dsRBP**	dsRNA binding protein
**ECM**	extracellular matrix
**EndMT**	endothelial to mesenchymal transition
**FGFR2**	fibroblast growth factor receptor 2
**FOXO3**	forkhead box O3
**FUS**	fused in sarcoma
**Fbln2**	fibullin-2
**HOTAIR**	HOX transcript antisense RNA
**HSRL**	human-specific regulatory loci
**lncRNA**	long non-coding RNA
**LOXL3**	lysyl oxidase-like protein-3
**MALAT1**	metastasis associated lung adenocarcinoma transcript 1
**Meg3**	maternally expressed gene 3
**METTL3**	methyltransferase like 3
**MIAT**	myocardial infarction-associated transcript
**MF**	myocardial fibrosis
**miRNA**	microRNA
**MMP2**	matrix metalloproteinase 2
**MMP9**	matrix metalloproteinase 9
**MMP14**	matrix metalloproteinase 14
**MDM2**	murine double minute 2
**mTOR**	mammalian target of rapamycin
**ncRNAs**	non-coding RNAs
**OGD**	oxygen and glucose deprivation
**PAMP**	pathogen-associated molecular pattern
**PHLPP2**	PH domain leucine-rich repeat protein phosphatase 2
**pre-miRNA**	precursor miRNA
**PTBP1**	polypyrimidine tract-binding protein 1
**PRG4**	proteoglycan 4
**RBP**	RNA binding protein
**RISC**	RNA-inducing silencing complex
**SEPP1**	selenoprotein P1
**Sfrp2**	secreted frizzled related protein 2
**SNX9**	sorting connexin 9
**SWI/SNF**	switch/sucrose non-fermentable
**TAGLN2**	transgelin 2
**TCF7**	transcription factor 7
**TIMP**	tissue inhibitors of matrix metalloproteinase
**TIMP1**	tissue inhibitors of matrix metalloproteinase 1
**TIMP4**	tissue inhibitors of matrix metalloproteinase 4
**TMP4**	tropomyosin-4
**TRPM7**	transient receptor potential melastatin 7
**VEGF**	vascular endothelial growth factor
